# A deep insight into the sialome of the house fly, *Musca domestica*, infected with the salivary gland hypertrophy virus (MdSGHV)

**DOI:** 10.1038/s41598-025-92569-6

**Published:** 2025-03-07

**Authors:** Stephen Lu, Noa Miller, Adrian Wilson, Christopher J. Geden, John G. Stoffolano, Jose M. C. Ribeiro

**Affiliations:** 1 Vector Biology Section, Laboratory of Malaria and Vector Research, National Institute of Allergy and Infectious Diseases, Bethesda, MD USA; 2 Stockbridge School of Agriculture, University of Massachusetts, Amherst, MA USA; 3 USDA/ARS Center for Medical, Agricultural, and Veterinary Entomology, Gainesville, FL USA

**Keywords:** Saliva, Arthropod, Vector, Transcriptome, Salivary gland, Sequence annotation, Entomology, Infectious diseases

## Abstract

The house fly, *Musca domestica*, serves as a mechanical vector for numerous pathogens, posing a significant risk to human and animal health. More than two decades ago, the *Musca domestica* salivary gland hypertrophy virus (MdSGHV) was discovered, infecting both males and females flies and disrupting mating and the reproductive process. While MdSGHV can infect various tissues, its primary replication site is the house fly salivary gland. It is well established that arthropod salivary glands play an important role not only in acquiring food but also in transmitting pathogens. Therefore, understanding the composition of vector salivary glands and the interactions between vector and pathogen components is essential for developing future control strategies. To this end, we conducted a comprehensive RNA-sequencing of salivary glands from both infected and non-infected house flies. Our analysis identified a total of 6,410 putative sequences, with 6,309 originating from *M. domestica* and 101 from the MdSGHV, categorized into 25 functional groups. Furthermore, differential expression analysis between infected and non-infected salivary glands revealed 2,852 significantly modulated transcripts, highlighting profound transcriptional changes triggered by MdSGHV infection. Overall, these findings not only deepen our understanding of the composition of *M. domestica* salivary glands but also provide valuable insight into the virus-vector interaction, which could serve as a model to understand other medically relevant interactions.

**Supplementary Information:**

The online version contains supplementary material available at 10.1038/s41598-025-92569-6.

## Introduction

The house fly, *Musca domestica*, is closely associated with humans and is a known vector for various pathogens, including bacteria, fungi, viruses, and protozoa^1^. Its capacity as a mechanical vector is primarily due its high mobility and its biological and ecological niches, which involves close proximity to decomposing matter such as manure, garbage, and carcasses. Flies can transmit pathogens by three different ways: mechanical dislodgment from their exoskeleton, fecal deposition and regurgitation^2^. During its lifespan, a single adult female can lay multiple batches of eggs, each containing 120 to 140 units, typically deposited on decomposing organic matter where larvae develop. The larvae progress through three instars before entering the pupal stage, which can last from a few days to up to 28 days depending on environmental conditions^3,4^.

Control of *M. domestica* primarily relies on insecticides, but the rapid development of resistance in house flies to every class of insecticides has made them a model for studying insecticide resistance^5,6^. The severity of the resistant problem prompted the use of alternative control strategies, including the use of parasitoid wasps for biological control^7,8^.

More than 20 years ago, house flies exhibiting hypertrophic salivary glands were identified and a double-strand DNA (dsDNA) virus was isolated from these glands, later named *Musca domestica* salivary gland hypertrophy virus (MdSGHV)^9^. In addition to causing salivary gland hypertrophy, MdSGHV infection has been shown to disrupt mating and reproductive behaviors in flies^10,11^, making it a potential candidate for biological control of flies^12^. Interestingly, surveys of wild house flies have revealed that the average prevalence of MdSGHV ranges from 0.5 to 10%, with some populations of house flies reaching as high as 34%^13,14^.

In addition to MdSGHV, other salivary gland hypertrophy viruses have been identified in the tsetse fly *Glossina pallidipes*^15^ and the narcissus bulb fly *Merodon equestris*^16^. All three viruses share rod-shape viral particles and a dsDNA genome, which lead to their classification into a novel virus family named *Hytrosaviridae*^17^. In the case of MdSGHV, viral particles have been found in various tissues, including the midgut, ovaries, fat body, crop, and brain. However, the primary site of viral replication is the house fly salivary glands^18^. Additionally, mature viral particles were detected in both oral secretions and excreta of the house fly^19^, suggesting that horizontal transmission among adult flies is the primary mode of infection. Notably, when flies were exposed to different contaminated substrates, infection rates varied, indicating that viral survivability and infectious capacity may depend on the type of substrate and possibly environmental conditions^14^. However, further studies are needed to better understand the exact mechanisms of MdSGHV transmission.

Given the critical role of the salivary gland in the virus-fly interaction, an ultrastructural study was conducted on infected and non-infected flies. The study revealed that the secretory portion of the house fly salivary gland is the most affected by MdSGHV infection^20^. Notably, the study described the presence of a chitinous lining the salivary duct in PBS-injected flies. However, this lining was absent in flies infected with MdSGHV. This observation led to the hypothesis that the degradation of this membrane could represent an important step for the virus to gain access to the saliva.

To enhance our understanding of the virus-fly interactions, we conducted extensive RNA-sequencing of infected and non-infected *M. domestica* salivary glands. Here we present a detailed characterization of *M. domestica* salivary gland composition and offers insights into the specific interactions between the virus and its vector in the salivary gland.

## Materials and methods

### House fly rearing, virus infection and salivary gland dissection

House flies were from the WHF strain maintained in CJG’s lab and were reared on a larval diet composed of wheat bran, pelleted calf feed, and water. Fly pupae were shipped overnight to JGS and kept in wire-screened cages along with a 500 cm^3^ container of water and a separate container filled with a 50:50 mixture of powdered whole milk and granulated sugar, fitted with a saturated Absorbal wick. Flies were reared in a controlled environment at 24–25ºC and 70% relative humidity under a 12:12 h light-dark photoperiod. After a 24-hour period following emergence, the adults were anesthetized with CO_2_ and subsequently placed on an ice-bath. Females were then intrathoracically injected with either 2.5 µl of PBS or with a MdSGHV preparation. The MdSGHV stock was prepared by combining 100 µl of infected salivary glands with 100 ml of PBS solution^21^. The groups of injected flies were then maintained for 5 days in vented plastic containers equipped with the same amount of food and water, after which their salivary glands were dissected. Three independent biological replicates were collected for both infected and non-infected samples. Each sample consisted of a pool of 65–70 *M. domestica* salivary gland pairs. Immediately following dissection, the salivary glands were immersed in 200 µl of RNAlater solution, kept at 4ºC for 48 h, and subsequently stored at -80ºC until further processing.

### Library preparation, sequencing and data analysis

Total RNA was isolated from three independent pools of PBS-injected or virus-infected salivary glands using the AllPrep DNA/RNA/Protein mini kit (QIAGEN) according to the manufacturer instructions. RNA integrity and quantification were assessed using a Bioanalyzer 2100 system (Agilent Technologies). The Illumina libraries were constructed using the NEBNextUltraTM II (Directional) RNA with polyA selection library prep kit and sequencing was performed in an Illumina Novaseq 6000 DNA sequencer. The quality of raw Illumina reads were checked using the FastQC tool (https://www.bioinformatics.babraham.ac.uk/projects/fastqc/). Low-quality sequences with a Phred quality score (Q) below 20 and the Illumina adaptors were removed using TrimGalore (https://github.com/FelixKrueger/TrimGalore). Subsequently, reads were merged and *de novo* assembled using Trinity (2.9.0)^22^, in single-stranded F mode, and ABySS (2.3.1)^23^ with k values ranging from 25 to 95, with increments of 10. The final assemblies were merged, and sequences sharing at least 95% identity were consolidated using the CD-HIT tool^24^. The DNA coding sequences (CDS) with an open reading frame (ORF) of at least 150 nucleotides were extracted based on BLASTp results from several databases, including a subset of the non-redundant protein database, the transcriptome shotgun assembly (TSA), and Refseq-invertebrate. The CDS were extracted if they covered at least 70% of a matching protein. Additionally, all ORFs starting with a methionine and with a length of at least 40 amino acids were subjected to the SignalP tool (V3.0). Sequences with a putative signal peptide were mapped to the ORFs, and the most 5’ methionine was selected as the starting point of the transcript^25^. The *de novo* CDS were then merged with those currently annotated in *M. domestica* reference genome (GCF_030504385.1)^26^ and those annotated in the *M. domestica* salivary gland hypertrophy virus genome (GCF_000879935.1). Relative quantification of each CDS was performed by mapping the trimmed Illumina reads to the final set of CDS using RSEM^27^ and CDS with a TPM ≥ 3 in at least one biological condition was extracted for downstream analysis. Functional annotation of the selected CDS was carried out using an *in-house* program that scanned a vocabulary of approximately 450 words and their order of appearance in the protein matches obtained from BLASTp/RPS-BLAST against various databases, including Transcriptome Shotgun Assembly (TSA), a subset from the Non-Redundant (NR), Refseq-invertebrate, Refseq-virus, UNIPROTKB, CDD, SMART, MEROPS, EC, and PFAM). This annotation process included percent identities and coverage information^28^. The final annotated CDS are available for download as a hyperlinked Excel file (Supplementary file 1).

### Statistical analysis

The multidimensional plot and the pairwise differential expression analysis were carried out with the edgeR package^29^ for R^30^, using the classical approach with the exactTest function and the PBS-injected samples as reference. Statistical significance was considered when LogFoldChange higher than 2 or lesser than − 2, alongside a false discovery rate (FDR) less than 0.05 were obtained. The heatmap plot was generated with the pheatmap (10.32614/CRAN.package.pheatmap) package using the TPM values and the volcano plots were generated with the ggplot2 (10.32614/CRAN.package.ggplot2) package for R.

## Results and discussion

### Overview of the sialotranscriptome of infected and non-infected *M. domestica*

Illumina sequencing of our six libraries of PBS-injected or infected-salivary glands from *M. domestica*, yielded a total of 295,399,368 high quality reads. From our *de novo* assembly strategy utilizing both ABySS and Trinity assemblers, 110,950 sequences were generated, from which 29,390 putative CDS were extracted. This set of CDS was subsequently merged with the 28,176 currently annotated in *M. domestica* genome and the 108 putative CDS from the *M. domestica* salivary gland hypertrophy virus genome. After filtering sequences with at least 95% of identity, we obtained 37,969 putative transcripts.

The relative abundance of each putative CDS was estimated by the transcript per million (TPM) parameters by mapping the trimmed Illumina reads to the 37,969 transcripts. Our mapping rates were consistent across the three independent biological samples for both PBS- and infected-salivary glands. Specifically, the PBS-injected libraries exhibited mapping rate of 36.77% ± 4.4%, while in libraries obtained from infected-salivary glands showed 46.62% ± 1.8%. These mapping rates align within those reported in other transcriptome studies focused on the salivary glands of arthropods^31–33^, emphasizing the overall consistency of our samples and indicating the absence of outliers. It is important to note that the unmapped reads could originate from 5’ and 3’ UTR regions, which were removed during our CDS extraction pipeline, as well as non-coding RNA or any potential sequence that eluded extraction due to lack of homology with previously deposited sequence or signal peptide.

For downstream analysis, we specifically focused on putative coding sequences with TPM values of at least three in PBS-injected or infected salivary glands. This yielded a total of 6,309 putative CDS from *M. domestica* and 101 CDS from the MdSGHV. Among these, 1,494 were detected in both PBS and infected samples, 651 were uniquely present in PBS-injected samples, and 4,265 were identified solely in infected salivary glands. As expected, none of the 101 CDS from MdSGHV were observed in our PBS-injected samples.

**Fig. 1:**
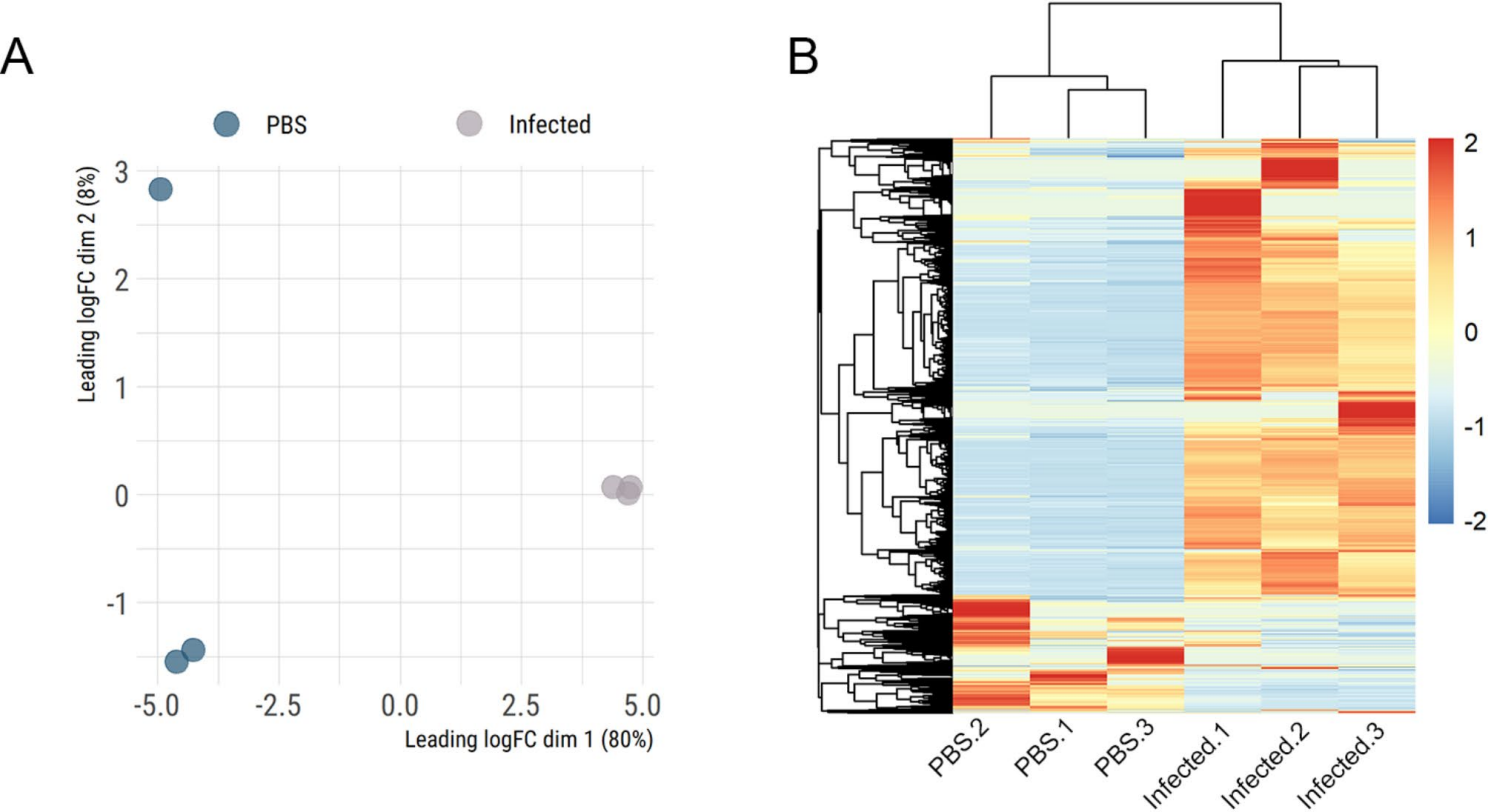
Overview of the RNA-sequencing from infected and non-infected salivary glands from *M. domestica*. (**A**) Multidimensional plot of the putative transcripts identified in *M. domestica* salivary glands with TPM ≥ 3. (**B**) Heatmap plot of the Z-score of the normalized TPM values of the 6,410 putative CDS with TPM ≥ 3.

Initial data exploration through dimensional analysis highlights the distinct transcriptional profile between PBS-injected and infected-samples (Fig. 1A). While one of our PBS samples appears to be an outlier, it is important to note that the y-axis only contributes to 8% of the variance between the samples. Consequently, this preliminary analysis suggests an overall similarity and high reproducibility of our samples. Furthermore, a heatmap plot, utilizing the normalized TPM values of each transcript, provides additional insight into the overall difference between infected and control groups (Fig. 1B), in which a dichotomic pattern between our two biological conditions was found. Specifically, the majority of the putative transcripts were found to be prevalent in infected samples, while a reduced number of CDS were found to be uniquely abundant in PBS-injected samples. These findings highlight the dramatic alterations in the transcriptional landscape of *M. domestica* salivary glands following MdSGHV infection.

**Fig. 2:**
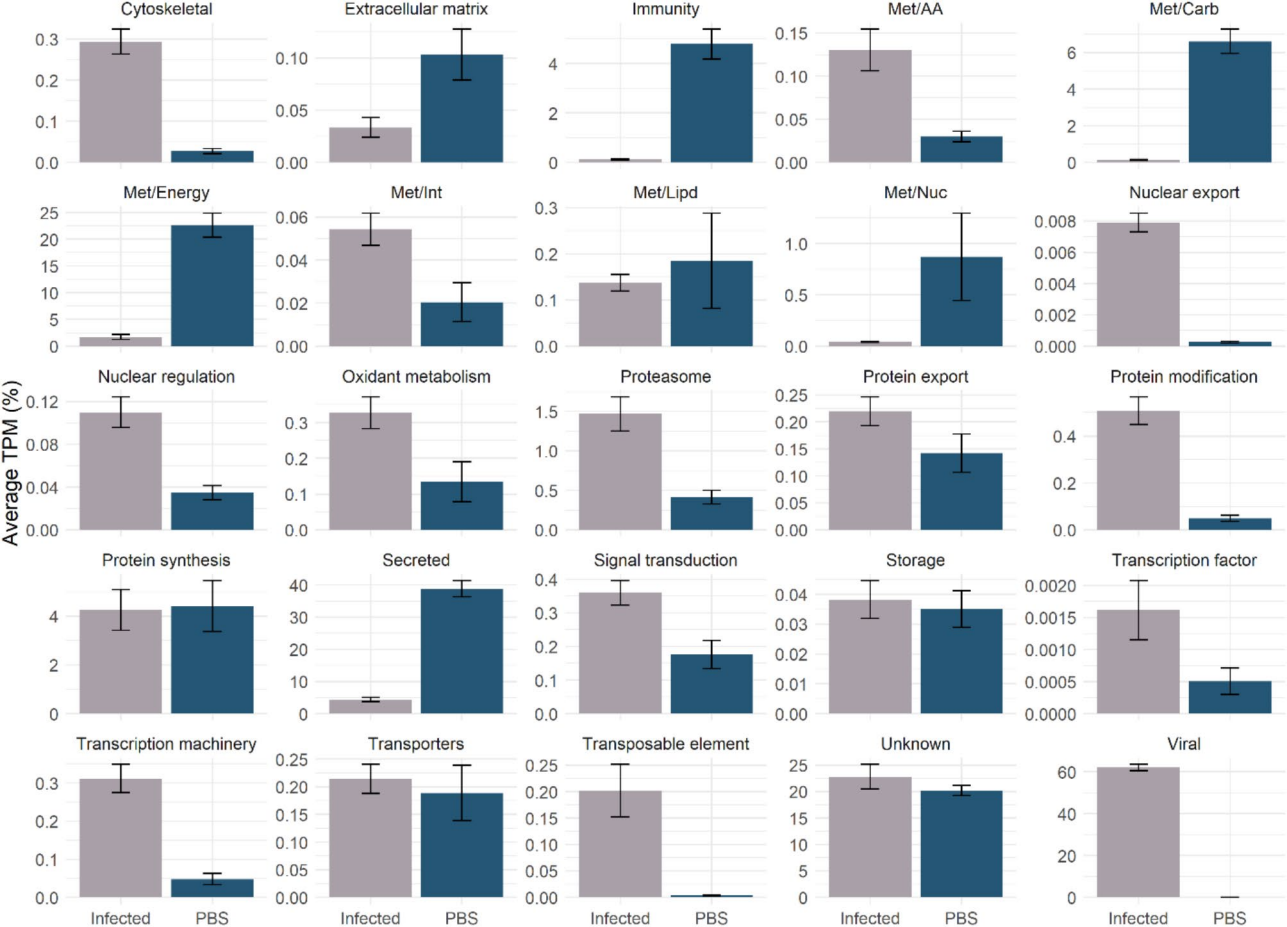
Functional classification of the 6,410 putative transcripts with TPM ≥ 3 identified in PBS- or infected-salivary glands from *M. domestica*. The bars represent the average TPM (%) of each class, while the error bars represent the standard deviation of the mean.

Our functional annotation analysis successfully classified the 6,410 putative transcripts into 25 classes (Fig. 2), where significant differences in the overall abundance of each class was evident between infected and non-infected flies (Fig. 2). Specifically, in PBS-injected samples the most prevalent functional groups were “secreted” (38.8%), “Energetic metabolism – Met/Energy” (22.6%) and “unknown” (20.2%). In contrast, infected samples exhibited a distinct pattern with “virus” (62.1%), “unknown” (22.8%) and “Secreted” (4.3%) being the most abundant classes. It’s quite remarkable that in our infected samples we observed that most mapped reads, approximately 60% (Fig. 2), were of viral origin, highlighting how MdSGHV can effectively hijack its host cellular machinery to replicate. Our functional annotation tool incorporates the “unknown” class, encompassing transcripts with either high coverage and identity with previously deposited sequence of unknown function or those exhibiting low identity values, and therefore, they can be considered potential novel sequences. It’s important to recognize that in both infected and non-infected samples the “unknown” functional group was among the most abundant ones, highlighting the current knowledge gap regarding salivary proteins from the house fly. Furthermore, the “secreted” class contains CDS bearing a putative signal peptide, representing proteins that are potentially secreted into *M. domestica* saliva. Within the “secreted” class, we encounter putative transcripts encoding acid phosphatase, amylases, maltases, antigen 5-like, cysteine and serine peptidases, odorant-binding proteins, and antimicrobial peptides (Table 1). Notably, such proteins families have often been described to be present in the salivary glands of other blood-feeding and non-blood feeding arthropods^34–36^. The curated set of CDS, along with their functional annotation, has been compiled into a Windows-compatible Excel spreadsheet (Supplementary file 1).

**Table 1:** Functional classification of the putative “secreted” transcripts identified in *M. domestica* salivary glands with TPM ≥ 3.

Protein family	Number of CDS		Average TPM*	
	PBS	MdSGHV	PBS	MdSGHV
Antigen-5	27	0	41,217.6	0.0
Apolipoprotein	4	4	10,015.6	56.8
Enzyme				
Amylase	8	5	35,064.9	186.9
Maltase	0	5	0.0	45.3
Acid phosphatase	1	1	3.6	7.0
Cysteine peptidases	5	9	124.4	309.0
Dipeptidyl peptidase	0	5	0.0	44.2
Metallo peptidase	1	1	4.9	9.2
Serine peptidase	17	11	562.4	88.2
Esterases	1	3	4.0	19.3
Lipase	6	4	2,530.6	34.6
Hormone-binding protein	8	1	89.8	26.3
Immunity				
Attacin	1	12	4.3	88.6
Lectin	11	12	46,579.4	164.2
Diptericin	4	4	44.2	206.3
Cecropin	1	0	16.4	0.0
Lysozyme	8	2	409.2	40.1
Defensin	10	9	540.5	447.4
Mucin	0	6	0.0	85.3
Odorant-binding protein	11	4	234.7	40.3
Peptidase inhibitor				
Kunitz	9	6	693.9	74.4
Kazal	3	2	560.7	80.1
Cystatin	6	6	453.9	111.6
Serpin	6	10	33.2	105.1
Trypsin-inhibitor like (TIL)	5	2	622.8	165.1
Unknown	387	533	324,510.9	38,133.9

*TPM transcript per million.

**Fig. 3:**
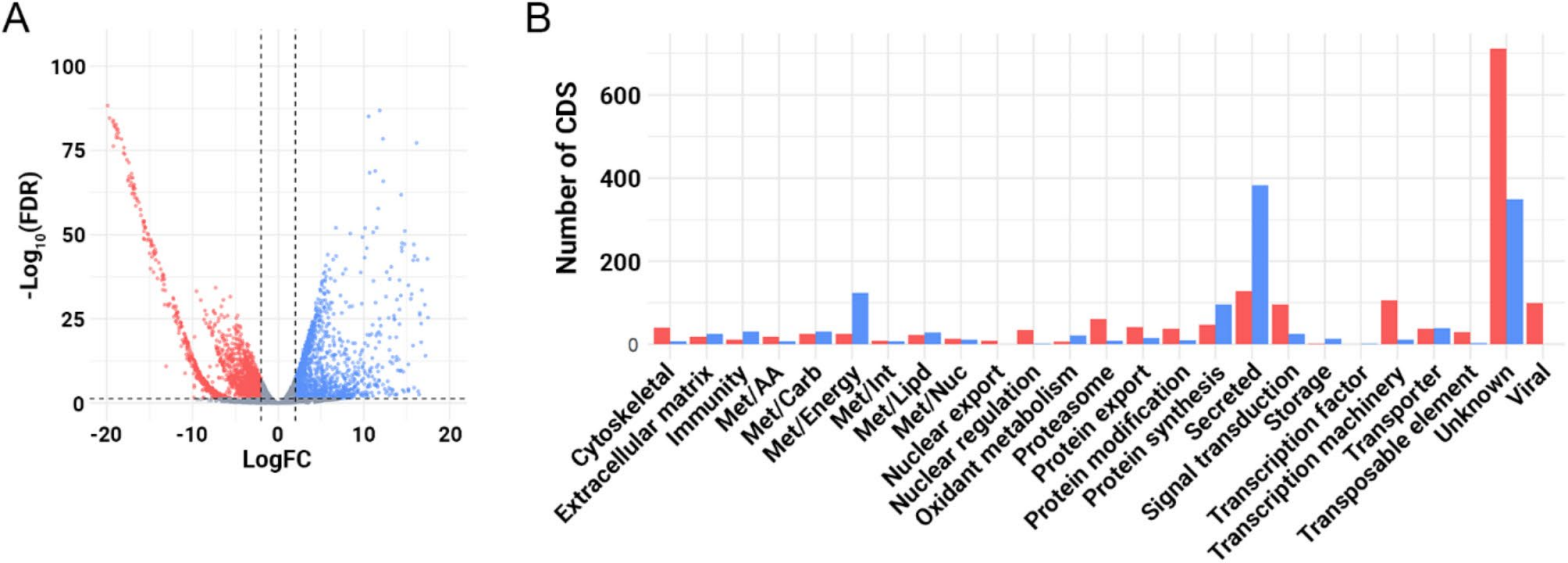
Differential expression analysis of the transcripts present in *M. domestica* infected and non-infected salivary glands. (**A**) Volcano plot highlighting the differentially expressed transcripts. Statistical difference was considered when a transcript presented a LogFC ≥ 2 or LogFC ≤ -2 (vertical dashed lines) and a False Discovery Rate (FDR) ≤ 0.05 (horizontal line). (**B**) Functional classification of the differential expressed transcripts. Up regulated transcripts in PBS-injected samples are shown in blue, while downregulated ones are shown in red. Transcript not considered differentially expressed are shown as gray.

Our differential expression analysis revealed the presence of 2,852 modulated putative transcripts, with 1,617 down- and 1,235 up-regulated (Fig. 3A). Further analysis of the functional classification of the differentially expressed transcripts revealed that the most affected classes are the “unknown” (1,149 CDS), “secreted” (509 CDS), “energy metabolism – met/energy” (149 CDS), “transcription machinery” (116 CDS) and “viral” (101 CDS) (Fig. 3B). Together, these observations underscore the changes in the transcriptional landscape of *M. domestica* salivary glands induced by MdSGHV.

While our transcriptomic analysis of infected and non-infected house flies provides valuable insights into the potential composition of the fly salivary glands and the transcriptional changes induced by MdSGHV infection, it is important to note that transcript abundance does not necessarily correlate with actual protein levels. Therefore, additional validation steps, such as proteomic studies, are essential to confirm the findings presented here. Moreover, our current dataset only reflects transcriptional changes at a single time point (5 days post-infection). Expanding this analysis to include additional time points will offer a more comprehensive, longitudinal understanding of the dynamic transcriptomic and proteomic landscape of *M. domestica* salivary glands during viral infection.

Since this is the first report focused on the composition of *M. domestica* salivary glands, we will provide a description of the main putative secreted protein families that potentially constitute the house fly saliva, based on our PBS-injected samples. This is followed by analysis of the differentially expressed transcripts triggered by MdSGHV infection.

### An insight into *M. domestica* secreted protein families

#### CAP superfamily

Members of the antigen 5-like proteins, alongside cysteine-rich secretory proteins and pathogenesis-related 1 proteins, constitute the CAP superfamily^37^. These proteins have been identified in various organism, including blood-feeding and non-blood feeding arthropods^38–41^ and are recognized as the major components of wasp venom^42^. In our current dataset, we identified 27 putative CDS encoding for antigen 5-like proteins, exhibiting a wide range of TPM values (3.0–17338.1). Notably, the transcript XP_058975013.1 was found to be the fourth most abundant transcript within the “secreted” class, with an average TPM of 17,338.1 (Supplementary file 1). Together, these transcripts represented 10.1% of all “secreted” CDS within the PBS-injected samples (Table 1), ranking the third most abundant protein family in PBS-injected samples.

While CAP superfamily members have been detected in diverse organisms, their exact function remains elusive, with only a few proteins functionally characterized. In snakes, they act as toxins, interfering with smooth muscle contraction^43^. In blood-feeding arthropods, salivary antigen 5-like proteins are suggested to aid in blood acquisition by disrupting the host hemostatic response. Notably, in triatomines, these proteins exhibit antioxidant properties and inhibit platelet aggregation^44^. Similarly, a salivary antigen 5-like protein from the horse fly, *Tabanus yao*, has been shown to block platelet aggregation and thrombus formation, following the incorporation of a disintegrin domain^45,46^.

Interestingly, several members of the antigen 5-like protein family have been identified in the salivary glands of male mosquitoes^36,40^, which primarily feed on sugary diets. Although functional studies are still lacking, it is plausible that these proteins are involved with their feeding behavior. Currently, no study on the potential role of antigen 5-like proteins have been conducted in *M. domestica*, however the high number of transcripts and their prevalence within the house fly salivary gland suggests an important role in its physiology.

#### Putative salivary enzymes

In our dataset, we have identified transcripts coding for various enzymes, including amylases, esterases, lipases, as well as serine, cysteine, and metallopeptidases (Table 1). Further examination of the cysteine peptidases revealed transcripts encoding cathepsins L, F and B. Among the serine peptidases identified, most were putative trypsin-like peptidases (Supplementary file 1). Notably, while most transcripts showed low TPM values, one putative amylase stood out, XP_005185288.1, which exhibited an average TPM of 14,292.8 ± 433.7 in our PBS-injected samples, making it the 12th most abundant transcript. Collectively the 5 putative amylases represent 5.29% of all “secreted” CDS, ranking as the fourth most abundant salivary protein family within the putative “secreted” CDS. It is worth noting that amylases and their enzymatic activity have been shown to be present in the salivary gland homogenates and saliva of blowflies^47^, mosquitoes^48,49^, and salivary gland homogenates of *M. domestica*^50^.

While we can infer the biochemical activity of enzymes based on functional annotation, their precise role in meal acquisition remains largely unknown. *M. domestica* primarily feeds on decomposing matter and possesses mouthparts adapted for consuming fluid or semi-fluid substrates, featuring a sponging proboscis^51^. Upon landing on a solid substrate, the house fly can deposit its saliva which assists in the breakdown and liquefaction of its food source. Subsequently, it begins to ingest the food-saliva mixture. It was also observed that houseflies can regurgitate its partially digested meal^3^, a behavior potentially linked to their role as vectors^1^. Considering the feeding behavior of *M. domestica*, it’s likely that the enzymes and peptidases identified in the current dataset are salivary components relevant for meal acquisition. By possessing various classes of enzymes targeting different macronutrients – such as amylases for carbohydrates, lipases and esterases for lipids, and serine, cysteine, metallo and dipeptidyl peptidases for proteins - *M. domestica* saliva can efficiently breaks down solid or semi-solid food resources^50^.

An additional facet of the salivary enzymatic activity lies in its involvement in the *M. domestica* crop. Previous observations indicate that during the onset of a meal, the fluid primarily enters the crop before gradually transitioning into the midgut^3^. Consequently, it is plausible that the catalytic activity exert by the identified putative enzymes persists as the meal-saliva mixture traverses into the crop, a phenomenon previously evidenced in blowflies^47^.

In our analysis of putative serine peptidases transcripts, we also identified those harboring a CLIP-domain (PFAM 18322). In arthropods, the prophenoloxidase pathway serves as an important component of the immune system^52^, with serine peptidases featuring the CLIP-domain playing a pivotal role in its activation^53^. Specifically, in *M. domestica*, the serine peptidase mdPAP1, containing the CLIP-domain, exhibited upregulation in larvae following bacterial challenge. Additionally, researchers conducted RNA interference knock down of mdPAP1, resulting in an overall reduction in the phenoloxidase activity and diminished survival rates of the fly during bacterial infection^54^. Therefore, besides their potential involvement in meal acquisition, some of the identified serine peptidases might also contribute to the house fly’s immune response.

Lastly, in recent decades, there has been a growing recognition of sequences bearing a structural resemblance to enzymes but lacking their catalytic residues^55^. Despite the absence of their expected catalytic activity, these enzymes have been found to have novel crucial physiological roles^56,57^. For instance, in fleas, numerous sequences encoding acid phosphatase-like proteins have been identified in their salivary glands. However, all these sequences feature substitutions in one or two catalytic residues^58^. Further biochemical assays confirmed that these salivary phosphatases lacked the typical catalytic activity found in members of this family. Instead, these salivary phosphatases demonstrated the ability to bind with high affinity to biogenic amines and leukotrienes, potentially influencing host homeostasis and immune responses, thereby facilitating blood acquisition^59^.

In our current dataset, we encounter a putative transcript encoding a serine peptidase akin to bovine chymotrypsin A (UNIPROT: P00766). However, XP_005178052.1 exhibited substitution in the residues constituting the conserved catalytic triad characteristic of this family. Both catalytic histidine and serine residues are replaced by arginine (Supplementary Fig. 1). Moreover, we have identified similar mutated serine peptidase sequences in other flies, indicating that this is not an artifact but rather a conserved sequence within the Cyclorrhapha taxonomic group. Additionally, the fly’s sequences exhibit an elongated C-terminal region compared to the bovine chymotrypsin A, which likely contributes to their novel activity.

#### Antimicrobial peptides

The analysis of *M. domestica* genome has revealed a notable increase in the number of putative genes associated with the fly immune system compared to the *Drosophila melanogaster* genome, particularly those involved in pathogen recognition and effector molecules^26^. Furthermore, previous research has functionally characterized several of these effector’s molecules at different stages of *M. domestica*^60–62^, underscoring the robustness of the house fly immune system. In *D. melanogaster*, the antimicrobial peptides are constitutively expressed in the salivary glands^63^, echoing findings in other arthropods where immune-related transcripts are consistently present in these glands and in some cases, in the arthropod saliva^36,41,64^.

Given this context, it is not surprising that we have identified numerous putative transcripts related to the immune system of the house fly in our current dataset. Among these, 11 putative transcripts encoding lectin-like sequence were found in our PBS-injected samples with high TPM values (> 3000) (Table 1). Lectins, belong to a superfamily of carbohydrate-binding proteins that play a critical role in pathogen recognition^65^. Additionally, despite being in overall low abundances, we uncovered multiple putative transcripts encoding antimicrobial peptides akin to attacin, diptericin, defensin, cecropin and lysozymes (Table 1). Together, this observation suggests the presence of bactericidal activity in *M. domestica* salivary glands and potentially, in its saliva.

Functionally, these salivary antimicrobial peptides likely regulate the population of microorganisms within the house fly’s diet, primarily composed of organic matter which can present high number of bacterial and fungi populations. Beyond this primary function, akin to salivary enzymes, these antimicrobial proteins may also contribute to maintaining crop health. Storage of the recent meal in the crop likely fosters favorable conditions for microbial growth, potentially posing risks to the house fly if left uncontrolled. Notably, in the mosquito *Aedes aegypti* it was demonstrated that a salivary bacteriolytic factor can be taken into the crop when mosquitoes fed on sugar^66^, emphasizing the potential significance of salivary proteins in the crop.

#### Odorant binding proteins (OBP)

Odorant binding proteins are small proteins characterized by a hydrophobic binding site and are commonly found in the salivary glands and sensory organs of arthropods. Within sensory organs, these proteins play an important role in chemoreception, aiding in the identification of hosts, potential mates, and food sources^67^. Despite their prevalence in arthropods, the precise function of salivary OBPs remains largely unknown. Notably, mosquitoes’ salivary glands and saliva harbor a prolific protein family known as D7, which are related to the OBP family^68^. Members of this family are often referred to as *kratagonists*(from the Greek “krato”, meaning “hold”, and agonists), and exhibit a notable ability to bind tightly to small agonists such as biogenic amines (e.g., serotonin, histamine and epinephrine) and leukotrienes^69^. Due to this chelating activity, it was hypothesized that salivary D7 proteins may assist in blood-meal acquisition by modulating host hemostatic and immune responses^70^.

In our current dataset, we identified 11 transcripts containing the putative odorant-binding domain (PFAM 01395) in our PBS-injected samples (Table 1). However, all 11 putative transcripts exhibited low TPM values, with average TPM values less than 100 (Supplemental file 1), suggesting an overall low abundance in the salivary gland and potentially in the saliva of *M. domestica*.

#### Peptidase inhibitors

In our current dataset we identified several transcripts coding for putative serine and cysteine peptidase inhibitors with overall low TPM values (average TPM < 600, supplementary file 1), suggesting that such proteins are not very abundant in the salivary gland, and potentially, in the saliva of *M. domestica* females. Within the serine peptidase inhibitors identified here, we observed transcripts belonging to the Kunitz, Kazal, Serpin and Trypsin-inhibitor like (TIL) families (Table 1).

In blood-feeding arthropods, the presence of salivary peptidase inhibitors is closely linked to the modulation of hemostatic and immune responses of their hosts, thereby aiding in the blood acquisition process^69^. For instance, in the black fly *Simulium guianense*, a salivary Kunitz-type inhibitor has been identified to effectively inhibit FXa and disrupt coagulation^71^. Similarly, in the mosquito *Aedes albopictus*, a serine peptidase inhibitor belonging to the serpin family has demonstrated the ability to inhibit FXa^72^. In *A. aegypti*, a salivary Kazal-type inhibitor has been found to interfere with thrombin activity^73^. It’s noteworthy that some peptidase inhibitors also possess antimicrobial properties. Ixodidin, a TIL-type inhibitor initially isolated from the hemolymph of the cattle tick *Rhipicephalus microplus*, has been shown to impede the growth of both gram-positive and gram-negative bacteria, although its molecular mechanism is currently unknown^74^. A second TIL-type inhibitor from *R. microplus*, named BmSI-7, was shown to inhibit subtilisin A and Pr1 peptidase from the entomopathogenic fungus *Metarhizium anisopliae*^75^, potentially contributing to the tick defense against fungi.

Currently, there is a lack of studies focused on the functional characterization of salivary proteins in the house fly, hence it is unknown if such salivary peptidase inhibitors display antimicrobial activity. However, considering the typical habit and food sources of *M. domestica*, it is conceivable that such inhibitors may play an important role inhibiting proteases from potential pathogens, such as bacteria and fungi, that are present in their meal. This inhibition could potentially regulate the growth of these pathogens, particularly during the storage period of the meal within the fly’s crop. Furthermore, these inhibitors may also serve as regulators of endogenous peptidases. It is well established that unchecked proteolytic activity can lead to significant damage to cells and tissues. Therefore, once ingested, these salivary inhibitors may play a role in regulating *M. domestica* peptidase activity within the crop.

In our dataset, Kunitz and TIL serine peptidase inhibitors were found to be the most abundant (Table 1). Upon closer examination of the primary sequence the Kunitz-type domain in *M. domestica*, we observed the characteristic 6-cysteine framework conserved in this inhibitor family (Supplementary Fig. 2). Although further biochemical assays are necessary, we can hypothesize the possible targets of such inhibitors based on their P1 residue^76^. In the case of seqSigP-3207, XP_005182678.1, and seqSigP-89,278 the presence of a P1 arginine suggests that these proteins likely target trypsin-like peptidases. Conversely, seqSigP-31,109 targets chymotrypsin-like peptidases, as indicated by the tyrosine in the P1 position, while XP_005182681.1 inhibits elastase-like peptidases, based on its alanine at P1 position. Similarly, when exploring *M. domestica* TIL-type inhibitors, Mdseq_54221 and seqSigP-46,504 likely inhibit chymotrypsin-like peptidases, whereas seqSigP-75,896 likely follows an inhibitory pattern similar to ixodidin^74^ (Supplementary Fig. 3).

#### Unknown secreted transcripts

As mentioned earlier, transcripts in this class include those with a putative signal peptide that either exhibited low identity and/or coverage compared to previously deposited sequences, or they have high identity to deposited sequences of unknown function. In our PBS-injected samples, this class contained the highest number of putative CDS (387) and was the most prevalent group in terms of average TPM value, constituting approximately 69.98% of all secreted transcripts (Table 1).

The transcript coding for the protein XP_058978242.1, was found to be the most abundant putative sequence within our entire dataset, boasting a TPM value of 137,690.3 ± 11,126.2 (Supplemental file 1). Analysis of the XP_058978242.1 sequence unveiled an open reading frame spanning 518 nucleotides, encoding a mature protein of 11.04 kDa rich in tryptophan and histidine. This sequence exhibited a notable abundance of negatively charged residues, with 13 glutamic acids and 9 aspartic acid residues, resulting in an isoelectric point of 4.44. Remarkably, the transcript coding for XP_058978242.1 appears to be a unique sequence from *M. domestica*, as BLASTp searches against several databases, including the most recent non-redundant database (search conducted on 06/10/2024), failed to retrieve significant matches. Furthermore, no conserved domains were identified when querying against the PFAM, CDD and SMART databases. Given its prevalence and uniqueness, XP_058978242.1 presents an intriguing candidate for further studies. Similarly, the transcript XP_005190356.2, the third most abundant transcript within our dataset (average TPM of 53,264.9) and the second most abundant “unknown secreted” CDS, also did not present any significant identity to previously deposited sequence, nor the presence of conserved domains (Supplementary file 1). Aside from its mature molecular weight of 10.86 kDa and isoelectric point of 4.51, the only distinct feature of XP_005190356.2 is the presence of 20 glycine residues, representing 19.6% of the amino acid composition of this putative sequence.

Overall, the high prevalence of this class underscores the existing knowledge deficit regarding the function of salivary proteins in the house fly. The abundance of these putative transcripts strongly implies their significant involvement in the physiological functions of *M. domestica* salivary glands. Moreover, their distinctiveness, apparent lack of identity to previously documented sequences, and the absence of conserved domains all suggest the presence of novel functionalities. Collectively, this data underscores the importance for more in-depth investigations aimed at elucidating the functional properties of salivary proteins across arthropods.

### Transcriptional changes induced by the MdSGHV

In our analysis comparing PBS- and MdSGHV-injected salivary glands, we detected significant changes in 2,852 putative transcripts (Fig. 3), highlighting the profound impact of the MdSGHV challenge. Among these, we identified 382 upregulated and 128 downregulated putative CDS within the “secreted” category in our PBS-injected samples, making it the second most affected class in our study (Fig. 3B). Further examination of the “secreted” protein families revealed a marked reduction in nearly all of them in our MdSGHV-injected samples in both the number of CDS identified and their average TPM value (Table 1).

Specifically, the antigen 5-like transcripts were absent in our infected samples, whereas they constituted the second most abundant protein family in our control groups. Exemplifying this marked difference, the transcript XM_005185994.4, encoding an antigen 5-like protein was the second most upregulated transcript in our PBS-injected samples, with a LogFC of 17.42 (Supplemental file 1). It is noteworthy that a salivary antigen 5-like protein from the mosquito *A. aegypti* was shown to bind to the Zika virus envelope protein with high affinity^77^. Hence, alongside their physiological contributions to the salivary gland physiology, it’s possible that *M. domestica* antigen 5-like proteins also play an important role during the infection and proliferation of MdSGHV.

The putative salivary enzymes were also significantly reduced in our MdSGHV samples. In our analysis we identified several transcripts encoding for putative amylases highly upregulated within our PBS-injected samples, with LogFC values ranging from 13.8 to 17.4 (Supplementary file 1). Overall, when taking in account the average TPM values, the amylase protein family presented a reduction of 99.4% in our infected samples in relation to the PBS-injected flies (Table 1), suggesting that the saliva of infected flies likely have a reduced enzymatic activity.

During infection, MdSGHV virions are detected in various organs of the house fly, such as the midgut, ovaries, fat body, crop, and brain. However, despite their presence, there is no evidence of viral replication in these organs and this phenomenon seems to be confined to the salivary glands^18^. The significant reduction in transcripts encoding putative secreted proteins in our infected samples suggests that MdSGHV can effectively hijack the cellular machinery of secretory cells within the salivary glands of *M. domestica*. This hijacking massively favors the production of viral particles over salivary proteins, likely leading to the disruption of the salivary glands’ morphology and less active saliva.

In addition to its impact on the reproductive behavior of *M. domestica*^10,11^, MdSGHV infection has been found to disrupt food consumption in both male and female house flies. Infected flies experienced significant reductions of up to 45% in carbohydrate intake and 62% in protein intake^78^. Similar adverse effects were observed in infected *G. morsitans centralis*. Infected tsetse flies exhibited increased probing attempts, prolonged feeding duration, and decreased blood intake compared to the control group^79^. Since arthropod saliva plays a vital role in acquiring a meal, the overall negative effect of the hypertrophy virus on fly feeding fitness may be partly due to the reduction in salivary components. In the case of *M. domestica*, the decreased catalytic activity of amylases, lipases, and serine peptidases potentially disrupts the liquefaction process of solid or semi-solid meals. It is noteworthy that this reduced feeding efficiency could also facilitate virus dissemination. Infected flies may take longer to complete their meals and likely secrete more contaminated saliva to compensate for its reduced enzymatic activity.

In our current dataset, we have observed a marked downregulation of immune-related transcripts in our infected samples. Specifically, transcripts encoding putative lectins exhibited LogFC values ranging from 11 to 14 (Supplementary file 1), indicating their high prevalence in our PBS-injected samples. Notably, there were mixed effects regarding putative antimicrobial peptides. While transcripts encoding lysozyme and cecropins were downregulated, we observed upregulation of attacin and diptericin-like sequences in our infected samples (Table 1). These findings collectively suggest that MdSGHV infection may disrupt the immune capabilities of *M. domestica’*s salivary glands and saliva. A previous transcriptome analysis, encompassing the entire body of infected and non-infected houseflies, identified several upregulated antimicrobial peptides, including diptericin, cecropins and attacins^80^. However, based on the data presented here, it is likely that the increase in some of these immune-related transcripts in the whole-body study is not attributable to changes in the salivary gland, but rather to immune-competent components such as hemocytes and the fat body of the fly.

When exploring the most downregulated transcripts in our PBS-injected samples, which encompasses those prevalent in the infected flies, we discovered a predominant presence of sequences from the MdSGHV. Among the 108 predicted MdSGHV ORFs^81^, we obtained reads that mapped to 101 of them, with ORFs 6, 7, 8, 9, 26, 60 and 61 being absent from our dataset (Supplementary file 1). This pattern mirrors findings from the whole-body transcriptome analysis of infected *M. domestica*, where the same viral putative ORFs were identified as the least abundant MdSGHV sequences, with read counts ranging from 4 to 47^80^. Similarly, the most abundant viral transcripts concur in both studies. We found ORFs 96, 86, 93, 48 and 37 to be the most abundant in our study, whereas in the whole-body study, ORFs 37, 86 and 96 were the most abundant^80^.

Although the complete MdSGHV genome is available since 2008^82^ and progress has been made in identifying its putative ORFs^81^, the functional characterization of viral proteins remains largely unexplored. Furthermore, most of the predicted 108 MdSGHV ORFs lack high identity to sequences of known function, and currently, only 21 of them possess a meaningful annotation based on homology (Table 2). One notable exception is MdSGHV ORF 96, which has been demonstrated to encode a protein associated with the viral envelope. Additionally, antibodies targeting MdSGHV96 have shown efficacy in reducing MdSGHV infectivity^83^, highlighting its importance for the viral life cycle. Hence, very little is known about the molecular mechanism underlying the entry and exit of viral particles from the salivary glands.

**Table 2:** Currently genome annotation for selected MdSGHV putative ORFs.

Accession number	Genome annotation	ORF
YP_001883329.1	DNA polymerase	1
YP_001883338.1	Mitochondrial carrier protein	10
YP_001883339.1	Dihydrofolate reductase	11
YP_001883340.1	Thymidylate synthase	12
YP_001883342.1	Molybdopterin oxidoreductase	14
YP_001883357.1	Pif-1	29
YP_001883361.1	Putative vacuolar sorting ATPase	33
YP_001883364.1	Matrix metalloproteinase	36
YP_001883367.1	p74	39
YP_001883375.1	Occlusion-derived virus envelope protein	47
YP_001883377.1	Ac150-like protein	49
YP_001883378.1	Aminoacylase	50
YP_001883380.1	dUTPase	52
YP_001883390.1	Ribonucleoside diphosphate reductase small subunit, rr2	62
YP_001883393.1	Ribonucleoside diphosphate reductase large subunit, rr1	65
YP_001883397.1	Thymidine kinase	69
YP_001883406.1	Inhibitor of apoptosis protein	78
YP_001883417.1	Pif-2	89
YP_001883432.1	Helicase-like protein	104
YP_001883434.1	per os infectivity factor-3	106
YP_001883436.1	Ac81-like protein	108

Recently, an ultrastructure study of *M. domestica* salivary glands revealed the presence of a chitinous lining alongside the salivary duct, effectively separating the secretory cells from the duct. However, after 3 to 4 days of MdSGHV infection, this cuticular lining was no longer present in the secretory region of the house fly salivary glands^20^. This observation prompted the hypothesis that degradation of the cuticle could be an important step for the virus to gain access to the house fly saliva. In our current dataset, we did not observe major differences in the putative transcripts encoding for the enzymes of the chitin biosynthesis pathway or in those encoding putative chitinases (Supplementary Fig. 4). This indicates that the expression level of key *M. domestica* transcripts related to chitin metabolism remain unaltered during MdSGHV infection in the salivary glands.

A second hypothesis is that viral proteins are directly associated with the degradation of the salivary duct cuticular lining. One such protein of interest, YP_001883364.1, contains the catalytic domain of the M10 metallopeptidase subfamily (Table 2). Metallopeptidases are known for their role in tissue remodeling^84^. Furthermore, homologs of metallopeptidases are commonly found in viruses belonging to the *Baculoviridae* family, which infect arthropods^85^. These enzymes have been suggested in disrupting the midgut basal lamina^86^, facilitating viral infection dissemination. Thus, YP_001883364.1 emerges as a promising candidate for further studies.

It is noteworthy that our annotation pipeline identified a CBM_14 chitin binding domain (PFAM 01607) in the viral transcripts YP_001883377.1 (Supplementary file 1), currently annotated as Ac150-like protein. Ac150-like sequences are common in baculovirus genomes, and knockdown studies with the *Autographa californica multiple nucleopolyhedrovirus*(AcMNPV) have shown that ΔAc150 strain exhibit reduced infection efficiency compared to the wild-type strain^87^. Although the AcMNPV Ac150 recombinant protein did not present chitin-binding activity^88^, it’s important to consider the differences presented between MdSGHV and AcMNPV sequences. AcMNPV Ac150-like encodes a smaller protein with a theoretical molecular weight of 11.16 kDa and pI of 4.55, whereas MdSGHV Ac150-like encodes a protein 19.03 kDa protein with pI 5.43. Despite both sequences containing the putative CBM_14 domain, predictions of their three-dimensional structure using AlphaFold revealed significant differences. In the MdSGHV sequence, two additional alpha-helices are observed near the chitin-binding domain, along with three-antiparallel β-sheet in its N-terminal region, features absent in the predicted structure of AcMNPV (Supplemental Fig. 5). Although biochemical analysis is pending, it is likely that MdSGHV Ac150-like protein possesses additional or different activities compared to those observed in the AcMNPV protein.

## Conclusion

While the importance of saliva in meal acquisition and processing is widely acknowledged, most studies on arthropod saliva and salivary gland have primarily focused on blood-feeding species. In our study, we provide a comprehensive analysis of the transcriptional composition of *M. domestica* salivary glands, highlighting the putative secreted proteins that likely constitute house fly saliva. Moreover, through a comparison of the transcriptome landscape between infected and non-infected flies, we demonstrate that MdSGHV infection induces significant transcriptional changes in the house fly salivary gland, potentially leading to alterations in saliva composition. Notably, MdSGHV hijacks its host salivary glands transcription machinery as reflected by 60% of the library reads mapping to viral transcripts. The findings presented here not only enhance our understanding of *M. domestica* salivary gland composition but also provide insight into the changes elicited by the MdSGHV challenge, enhancing our understanding of virus-vectors interactions.

## Electronic supplementary material

Below is the link to the electronic supplementary material.

Supplementary Material 1

## Data Availability

The transcriptome data was deposited to the National Center for Biotechnology Information (NCBI) under Bioproject PRJNA1119442 and BioSample accession SAMN4165338 - SAMN4165343. The raw reads were deposited to the Short Reads Archive of the NCBI under accessions SRR29263970 - SRR29263975. Putative transcripts originated from our de novo assembly were deposited in the Transcriptome Shotgun Assembly (TSA) database of NCBI under accession number GKWL00000000. All supplementary files can be downloaded as a single compressed (.zip) file from the link: https://proj-bip-prod-publicread.s3.amazonaws.com/transcriptome/Mdomestica_2024/MdSupplementaryFile1.zip.
