# Supplementary material for: A deep insight into the sialome of the house fly, Musca domestica, infected with the salivary gland hypertrophy virus (MdSGHV)
Source: Sci Rep. 2025 Mar 7;15:8047. doi: 10.1038/s41598-025-92569-6 (PMC11889141; doi:10.1038/s41598-025-92569-6)
Supplement: Supplementary file 1 — Supplementary Material 1 [file 41598_2025_92569_MOESM1_ESM.pdf]

```
                          10        20        30        40        50        60        70        80
                  ....|....|....|....|....|....|....|....|....|....|....|....|....|....|....|....|
P00766            CGVPAIQPVLSGLSRIVNGEEAVPGSWPWQVSLQD------KTGFHFCGGSLINENWVVTAAHCGVTTSDVVV-------
XP_055858327.1    ---------------------GYSQQPEYQVSLVVKLPGNYGDLIHLGNGVILDERLILTTADCLHFKFGP-NAKPTQLD
XP_055919007.1    ------------------------QQPPYQVSIILKMPGNFQN-PHLCNGVIIHEKLILTTADCMHFKFGM-TGPSNKLE
XP_037894553.1    ------------------------------VSIYLNSNQN-GPPVLVCNGVIIQCRLVLTIASCIHYQLTA-ESAAEPFQ
KAI8119276.1      ------------------------NQPSYQVSLYVHLHPR-GPPIHLCNGIIVQSRLILTTASCVHYKFSSSNSPVIPIA
XP_065356850.1    ------------------------NQPSYQVSLYVHLHPR-GPPVHLCNGIIVQSRLILTTASCVHYKFSSSNSPVIPIA
XP_005178052.1    ------------------------DQPSYQVSLHVNLHPK-GPPVHLCNGEIIQSRIIVTTARCVHYKFSP-NSPAVPLP
XP_061390518.1    ------------------------DQPSYQVSLHVNLHPK-GPPIHLCNGAIIQSRVIVTTAQCVYYKFSP-NSAAVPLP

                          90       100       110       120       130       140       150       160
                  ....|....|....|....|....|....|....|....|....|....|....|....|....|....|....|....|
P00766            --AGEFDQGSSSEK--IQKLKIAKVFKNSKYNSLTINNDITLLKLSTAASFSQTVSAVCLPSASDDFAAGTTCVTTGWGL
XP_055858327.1    PSVLSVFAGSTNGQTRALQMNVSSIEFEKNFNSTTLENDLALLTLEHPLPLGTRSDIRWIM-LDDNSHMNRSCLVNF--F
XP_055919007.1    ASAINVIAGSKTAQQDVIQMNVSSIELPTNFEPTTLENDLALLTLSEALPLETRFDIRWIM-IDDNTHVNRSCLVNF--Y
XP_037894553.1    SNKLFIIAGTSSDFASDLTVQVLDIIIANSFNVTTNENDLAILRLNSNLPLGIRNDLKWVI-LDDIDNADRPCLANF--Y
KAI8119276.1      PSMISVISGSTTTFREELTLQVSDIFVADNFNYTTGENDLAVLRLKGILPLDTRSDMSWIT-LADDEDFEGSCLANF--Y
XP_065356850.1    PSMISVISGSSTVFNEELTLQVSNIFVVDDFNYTTGENDLAVLRLNGILPLDTRNDMSWIT-LADDEDYDGSCMANF--Y
XP_005178052.1    ASALRVIAGSSTEFYDELIVGVTDVLVAKDFNYTTGENDLSLLRLSKTLPLDVRADMSWIT-LDDAANFEGPCVANY--Y
XP_061390518.1    PSALTVIAGSSTEFYDELTVGIKDVLIAKDFNYTTGENDLALLRLSKTLPLDVRADMSWIT-LDDAVNFEGPCVANY--Y

                         170       180       190       200       210       220       230       240
                  ....|....|....|....|....|....|....|....|....|....|....|....|....|....|....|....|
P00766            TRYTNANTPDRLQQASLPLLSNTNCKKYWGTK------------IKDAMICAGASGV-SSCMGDSGGPLVCKKNGAWTLV
XP_055858327.1    SRNKKDAIPDFYQTEELYMINNAICRNSTKYPAARKHDICSNYILPHRFDCEKANSF-LSLNNDRGTGLVCKSFLVGLLS
XP_055919007.1    TRNPNDAITDYYQTEELYTVNNDVCRNFTKYPAARKRDICSFYVLPHQYDCIKADSY-LSFNNDRGTALVCKNFLVGLLS
XP_037894553.1    FRNKLTKIPIHTRTEQLPLLPNSECHSISQFPLARRNDICSLYMLPCGFHCTTFDDFARRYNIDRGIGLLCKNHLVGLLS
KAI8119276.1      VRNSLNGAPNYIQSEHLPILDSEACGATSQYSNERENDICSFYYLPAGFNCNAVESL-QNYNGDRGTGLVCGNKLMGILS
XP_065356850.1    VRNSLDGAPNYIQSEQLPLLDSYSCGSKFEFPNQRENDVCSFYYLPAGFNCNAVETL-QNYNGDRGTGLVCGNKLMGLLS
XP_005178052.1    IRNSISTVPNYIQTEELPLLDAKKCQSHNLYSLVRDYDICSLYMIPLGLTCQALSTL-DHHNGDRGTGLVCENKLVGLLS
XP_061390518.1    IRNSISTVPNYIQTEELPLLDVKKCQSQNLYSQIRNYDICSLYMIPLGLSCQVSSTS-HTY-------------IFYIRF

                         250       260       270       280       290       300       310       320
                  ....|....|....|....|....|....|....|....|....|....|....|....|....|....|....|....|
P00766            GIVSWGS---STCSTS-TPGVYARVTALVNWVQQTLAAN-----------------------------------------
XP_055858327.1    SVVTRPNPA-NNCSDVVLKAFYTYINPHLMWIYSVMSKMESE------YI-STPYDASLNPLV----LNPIPSTDFSNSF
XP_055919007.1    SVVTRPNPV-NNCSDVVLRAIYSNIIPHLRWIYSVMSKMETQ------YI-STPYDASVKPLM----AIPNSAANITNT-
XP_037894553.1    TILPMPNANNFNCSQKILQSYYTNLEYHLTWIYNVIHTEELQVLKEGTYTASSPYDGLREEYPLL------------LND
KAI8119276.1      TILPLQNK--TDCLEKPIKAYYTAIIPNIGWIFDVITEEDLNMLNDGEYISSSPYAGETNAFVEPQVTEPEPTLVYSQLA
XP_065356850.1    TILPLQNK--TDCMEEPIKAYYTALVPHLAWIFDVITEEDLNMLNEGEYISSSPYAGETNAFVEPQVTEPATTLAYTEPF
XP_005178052.1    TILPKENSSDTHCSDGPINAFYANLAPHLGWIFDVISDEDLKMYDNGEFVSSLPYSGENGSIEEPNISEPGTISPLVGDN
XP_061390518.1    YIETSDNSSESYCSDGPINAFYTNLAPQLGWIFDVISDEDLKMYENGEFVSSLPYSGENGSIEEPNLTEPGTTSPLVGNN

                         330       340       350       360       370       380       390       400
                  ....|....|....|....|....|....|....|....|....|....|....|....|....|....|....|....|
P00766            --------------------------------------------------------------------------------
XP_055858327.1    NSTSTTNSTSITNSTSITNSTLLLNSTISTFPFTNLTMNST------------------VVNSTPNPLSTNSTKWNTTST
XP_055919007.1    -------------SQTASNSTILSNTTVSGFPFTNLTIPRNTSILNSTQHNSTYPNSTILNYATPHPLAFNSTKWNTTSI
XP_037894553.1    SIQMCNG----GNSAKAAS--------HVEWQFPIYGI---LVILYCGNN------------------------------
KAI8119276.1      NITVPTES-SVQNSATKNIKGNF-K--ILSLIFGIYFI---IQVLNC---------------------------------
XP_065356850.1    SITASTEV-SAQYSAAANVKDNF-K--LFSMIFGTYFI---VQALNC---------------------------------
XP_005178052.1    -VTSTTEPNDLPNEASNHVHGSL-TLAVATFILALFSI------------------------------------------
XP_061390518.1    -VTSTTESNDLSNAASSFIHASL-TATVATCILAIQFM---GYI------------------------------------

                         410       420       430       440       450       460       470
                  ....|....|....|....|....|....|....|....|....|....|....|....|....|....|
P00766            ---------------------------------------------------------------------------
XP_055858327.1    SIPVRNNTLKPITYNNVASTVRVPGYGSPPDTNIRHPSSKPPIQNIFKNRGVMVRLNYGSFMIAFAFLIVPFIYL
XP_055919007.1    GNPSRNQTTKPVLFNNGVSTGPVHSYGTSIDPHIKHPTPKPAVQNIFTNSGVLAKLSAGSLLILLAYFRLITFYL
XP_037894553.1    ---------------------------------------------------------------------------
KAI8119276.1      ---------------------------------------------------------------------------
XP_065356850.1    ---------------------------------------------------------------------------
XP_005178052.1    ---------------------------------------------------------------------------
XP_061390518.1    ---------------------------------------------------------------------------
```

Supplementary figure 1: Amino acid alignment of mature chymotrypsin A (P00766) from *Bos taurus* with putative serine peptidases-like sequences from *Episyrphus balteatus* (XP_055858327.1), *Eupeodes corollae* (XP_055919007.1), *Glossina fuscipes* (XP_037894553.1),

Lucilia cuprina (KAI8119276.1), *Calliphora vicina* (XP_065356850.1), *Musca domestica* (XP_005178052.1), and *M. vetustissima* (XP_061390518.1). The residues that form the catalytic triad, His^{75}, Asp^{120} and Ser^{213} (numbering based on P00766 sequence) are highlighted in red.

```
                           10        20        30        40        50
                   ....|....|....|....|....|....|....|....|....|....|
BPTI               TPGCDTSNQAKAQRPDFCLEP------PYTGPCKARIIRYFYNAKAGLCQ
seqSigP-3207       ------------FDKADCSLPKD------VGPCRKADLSYYFDTESKSCQ
XP_005182683.1     ------------LKDAVCGQAHAKNGN-GVISCLGFMKSWSYNVESNECT
XP_005182678.1     ------------LKDPICGQPSAVIG-----ICRAEIPKFTYNAASNECI
seqSigP-31109      ------------LKNTICGLEHSKTTNSAGASCYAMIPSWSYNADAKECI
seqSigP-89278      ------------LKDPICGLPHSQDG-DNDLQCRGRFPLFSYNSDAKECV
XP_005182681.1     ------------LKHDICGLKHSKDGNDNGLACAAYFPSWSYKADTNECV

                           60        70        80
                   ....|....|....|....|....|....|....
BPTI               TFVYGGCRAKRNNFKSAEDCMRTCGGAIGPWENL
seqSigP-3207       TFFYGGCHGNNNRFNSKEECEKSCL---------
XP_005182683.1     EFVYGGCMGNDNRFESKEACEQKCKE--------
XP_005182678.1     SFVYGGCHGNDNNFATKEECEEKCKE--------
seqSigP-31109      HFVYGGCNGNENRFRTKEECLEMCAE--------
seqSigP-89278      SFIYGGCGGNDNRFSSKELCEEKCKE--------
XP_005181681.1     EFVYGGCGGNDNRFFSKEECEAKCKE--------
```

Supplementary figure 2: Amino acid alignment of complete and mature *M. domestica* putative transcripts encoding for Kunitz-type serine peptidase inhibitors and the bovine pancreatic trypsin inhibitor (BPTI, GeneBank: P00974.2). The conserved 6-cysteine framework from the Kunitz-domain is highlighted in blue and the putative P1 residue is highlighted in red.

```
                   10        20        30        40        50        60
               ....|....|....|....|....|....|....|....|....|....|....|....|
Ixodidin       QRGSRGQRCGPGEVFNQCGSACPRVCGRPPAQACTLQCVSGCFCRRGYIRTQRGGCIPERQCHQR
Mdseq_54221    --APPREACGENEEFVTCGTSCPLTCEKLTPGICTYQCFIGCQCKDGFFRNSEYKCVAETEC---
seqSigP-46504  --APPREACGENEEFVTCGTSCPLTCEKRTPGICTYQCFIGCQCKDGFFRNSEYKCVAETEC---
seqSigP-75896  ----APQECGENQEFTTCGTACPLKCNTPEPSFCTLQCVIGCQCKQGYRLNDSGACVLTKDC---
```

Supplementary figure 3: Amino acid alignment of complete and mature *M. domestica* putative transcripts encoding for trypsin-inhibitor like (TIL) serine peptidase inhibitors and Ixodidin from the cattle tick *R. microplus* (GeneBank: P83516.2). The conserved 10-cysteine framework from the TIL-domain is highlighted in blue and the putative P1 residue is highlighted in red.

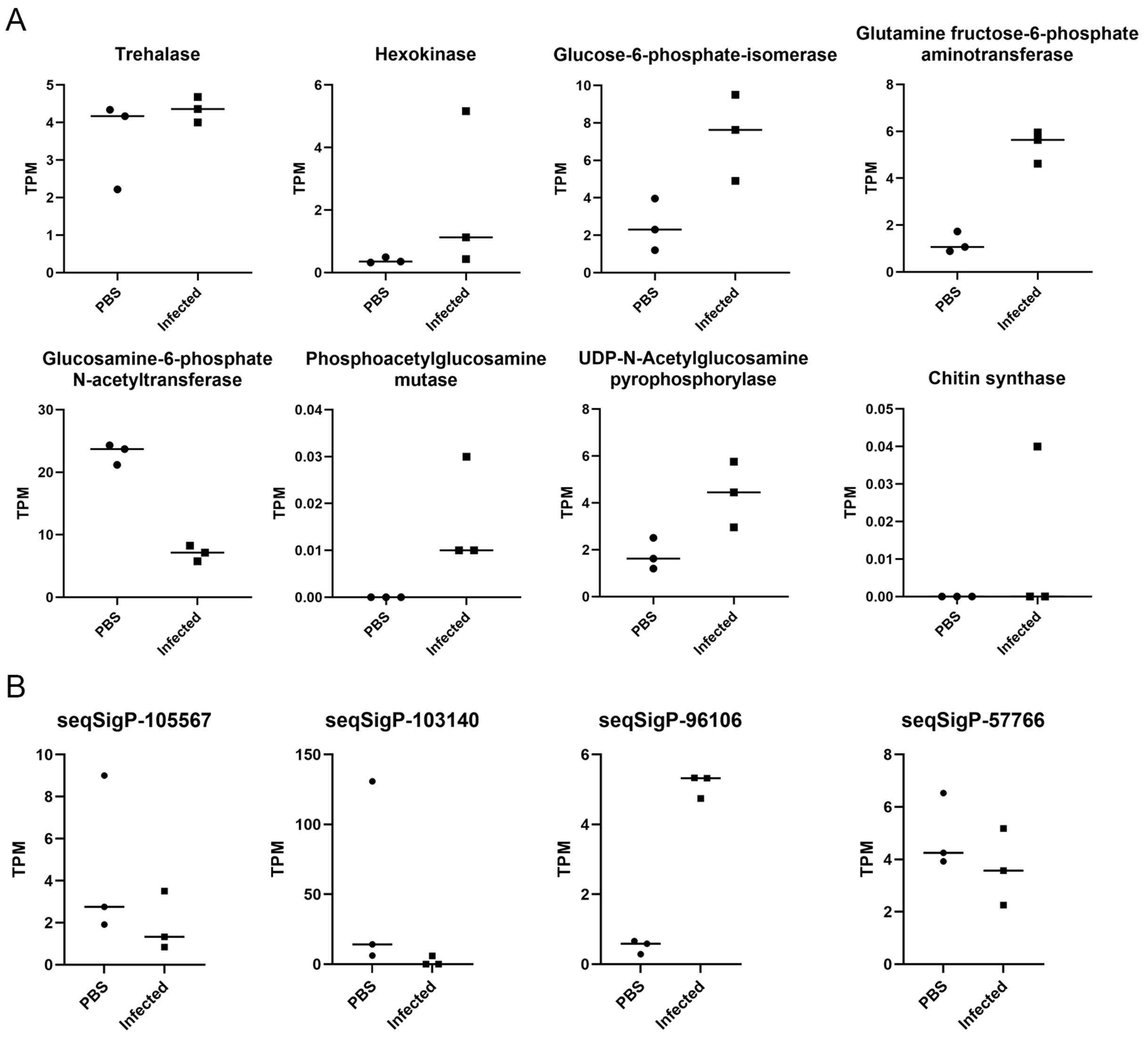

Supplementary figure 4: TPM values of putative transcripts encoding (A) enzymes associated with chitin biosynthesis and (B) chitinase-like sequences identified in the salivary gland transcriptome of *M. domestica* injected with PBS or infected with the MdSGHV. Each dot represents the TPM value identified in each biological sample, and the dash indicates the average value.

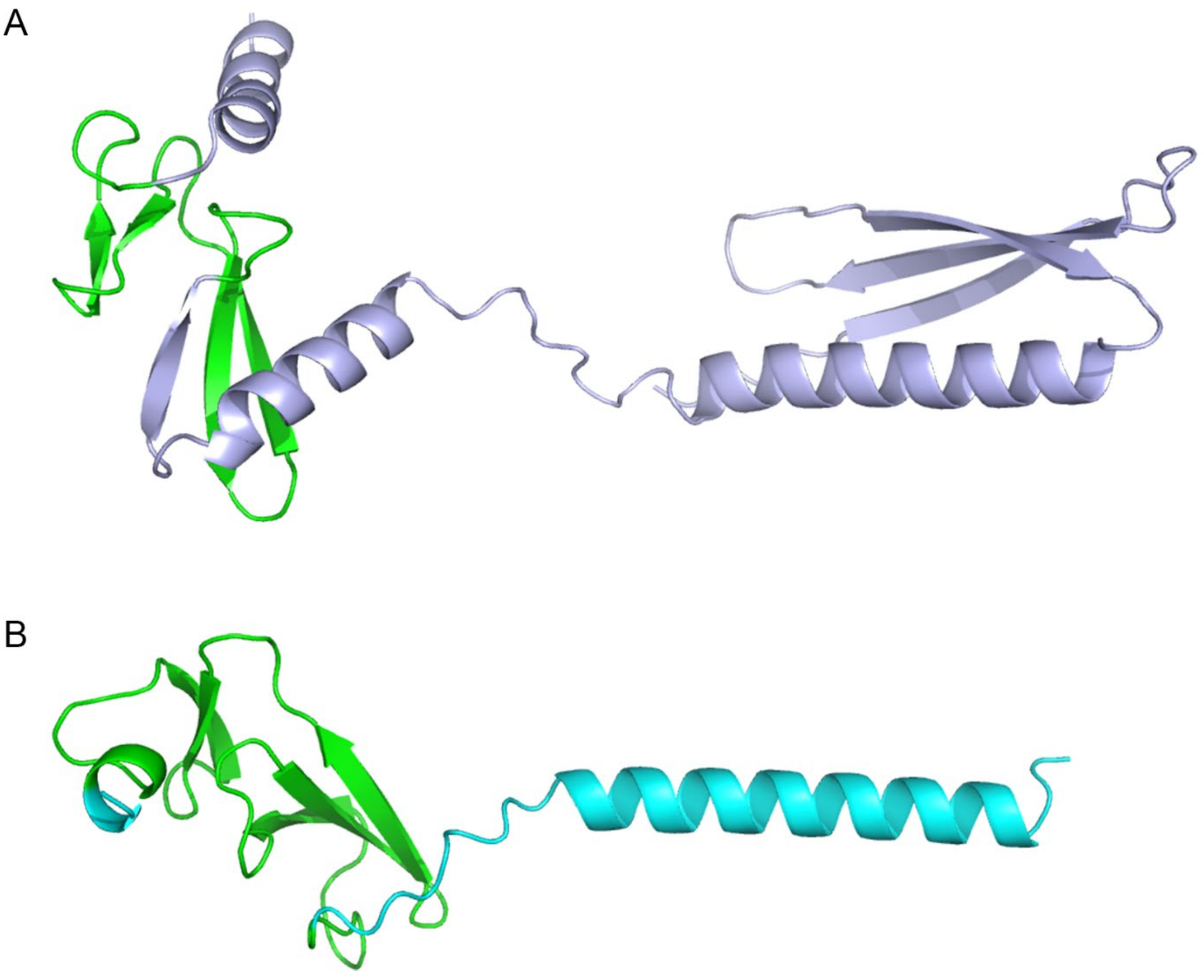

Supplementary figure 5: Predicted tridimensional structure model by AlphaFold of (A) MdSGHV Ac150-like and (B) *Autographa californica multiple nucleopolyhedrovirus* (AcMNPV) Ac150-like sequence. The CBM_14 chitin biding domain is highlighted in green.

Supplementary file 1: Windows-compatible hyperlinked Excel and associated files containing the functional annotation from the 6,945 CDS with a TPM of at least 3 identified in *M. domestica* salivary glands.
